# Genetic and environmental factors and serum hormones, and risk of estrogen receptor-positive breast cancer in pre- and postmenopausal Japanese women

**DOI:** 10.18632/oncotarget.20182

**Published:** 2017-08-11

**Authors:** Jiazhi Guo, Aiko Sueta, Koshi Nakamura, Nobuyasu Yoshimoto, Motoi Baba, Naoko Ishida, Kanako Hagio, Tatsuya Toyama, Hirotaka Iwase, Akiko Tamakoshi, Hiroko Yamashita

**Affiliations:** ^1^ Department of Breast Surgery, Hokkaido University Hospital, Sapporo, Japan; ^2^ Department of Breast and Endocrine Surgery, Kumamoto University Graduate School of Medical Sciences, Kumamoto, Japan; ^3^ Department of Public Health, Hokkaido University Graduate School of Medicine, Sapporo, Japan; ^4^ Department of Breast Surgery, Nagoya City University Graduate School of Medical Sciences, Nagoya, Japan

**Keywords:** breast cancer, estrogen receptor-positive, risk predictor, genetic variants, 25-hydroxyvitamin D

## Abstract

Breast cancer incidence in Japanese women has more than tripled over the past two decades. We have previously shown that this marked increase is mostly due to an increase in the estrogen receptor (ER)-positive, HER2-negative subtype. We conducted a case–control study; ER-positive, HER2-negative breast cancer patients who were diagnosed since 2011 and women without disease were recruited. Environmental factors, serum levels of testosterone and 25-hydroxyvitamin D, and common genetic variants reported as predictors of ER-positive breast cancer or found in Asian women were evaluated between patients and controls in pre- and postmenopausal women. To identify important risk predictors, risk prediction models were created by logistic regression models. In premenopausal women, two environmental factors (history of breastfeeding, and history of benign breast disease) and four genetic variants (TOX3-rs3803662, ESR1-rs2046210, 8q24-rs13281615, and SLC4A7-rs4973768) were considered to be risk predictors, whereas three environmental factors (body mass index, history of breastfeeding, and hyperlipidemia), serum levels of testosterone and 25-hydroxyvitamin D, and two genetic variants (TOX3-rs3803662 and ESR1-rs2046210) were identified as risk predictors. Inclusion of common genetic variants and serum hormone measurements as well as environmental factors improved risk assessment models. The decline in the birthrate according to recent changes of lifestyle might be the main cause of the recent notable increase in the incidence of ER-positive breast cancer in Japanese women.

## INTRODUCTION

Breast cancer is the most common cancer in Japanese women as well as in women worldwide. Its incidence in Japanese women has more than tripled over the past two decades (Cancer Registry and Statistics. Cancer Information Service, National Cancer Center, Japan, http://ganjoho.jp/en/index.html). We have previously shown that this marked increase in breast cancer incidence is mostly due to an increase in the estrogen receptor (ER)-positive, HER2-negative subtype [1, 2]. The rate of ER-positive breast cancer is reported to be approximately 90% among cancer patients in their 40s, and approximately 80% in those aged 50 years or older in 2011 in Japan [2]. Risk assessment tools have been used to predict the risk of breast cancer in Western countries, and prevention trials have shown that tamoxifen and aromatase inhibitors lower ER-positive breast cancer incidence in women determined to be at increased risk based on the Gail model and the Tyrer-Cuzick model [3–5].

The penetration of risk factors may vary by breast cancer subtype, especially those defined by ER status, and ethnicity [6, 7]. The reported risk factors can be divided into three categories; environmental factors, endogenous factors including hormones, and common genetic variants including single nucleotide polymorphisms (SNPs) [8]. Previous studies demonstrated that inclusion of common genetic variants as well as environmental factors could improve risk assessment models [9–12]. In addition, since there are bimodal premenopausal and postmenopausal breast cancer populations [13], the etiology of pre- and postmenopausal breast cancers is likely to be different, especially in ER-positive breast cancer [14]. Establishment of risk factors, both genetic and environmental, capable of predicting the risk of ER-positive breast cancer, which will enable the efficient selection of candidates for preventive therapy, is urgently needed in Japanese women.

We previously analyzed genetic and environmental factors, including 14 SNPs, serum levels of circulating hormones and growth factors, and mammographic density among breast cancer patients and controls, and created risk prediction models for ER-positive breast cancer [9]. In this case–control study, breast cancer patients diagnosed since 2011 were recruited in order to reflect the recent marked increase in the incidence of ER-positive breast cancer, and created improved risk prediction models to identify important risk predictors.

## RESULTS

### Environmental factors

In premenopausal women, younger age (*P* = 0.015), a lower number of pregnancies (*P* = 0.003), nulliparity (*P* = 0.004), a history of never breastfeeding (*P* = 0.001), and presence of a family history of breast cancer (*P* = 0.034) were observed in patients compared to controls (Table 1). On the other hand, older age (*P* < 0.001), body mass index (BMI) ≥ 25 kg/m^2^ (*P* < 0.001), older age at menarche (*P* < 0.001), a history of never breastfeeding (*P* = 0.009), presence of hyperlipidemia (*P* < 0.001), and presence of diabetes mellitus (*P* = 0.029) were found in postmenopausal patients compared to control women (Table 1).

**Table 1:** Distribution of environmental factors between patients and controls

	Premenopausal women			Postmenopausal women		
	Patients (*n* = 103)	Controls (*n* = 303)	*P*-values	Patients (*n* = 150)	Controls (*n* = 602)	*P*-values
Age (years)						
40–49	75 (72.8%)	180 (59.4%)	0.015*^a^			
50–59	28 (27.2%)	123 (40.6%)		38 (25.3%)	219 (36.4%)	< 0.001*^a^
60–69				72 (48.0%)	311 (51.7%)	
70–79				40 (26.7%)	72 (12.0%)	
Mean ± SD	47.0 ± 3.8	48.7 ± 2.5	< 0.001*^b^	64.6 ± 7.2	61.5 ± 6.3	< 0.001*^b^
Body mass index (kg/m^2^)						
< 25.0	79 (76.7%)	242 (79.9%)	0.50^a^	103 (68.7%)	501 (83.2%)	< 0.001*^a^
≥ 25.0	24 (23.3%)	61 (20.1%)		47 (31.3%)	101 (16.8%)	
Mean ± SD	23.0 ± 3.9	22.5 ± 3.9	0.28^b^	23.5 ± 4.2	22.2 ± 3.0	< 0.001*^b^
Age at menarche (years)						
≤ 12	55 (53.4%)	179 (59.1%)	0.59^a^	35 (23.3%)	244 (40.5%)	< 0.001*^a^
13–14	40 (38.8%)	102 (33.7%)		74 (49.3%)	241 (40.0%)	
≥ 15	8 (7.8%)	22 (7.3%)		41 (27.3%)	117 (19.4%)	
Mean ± SD	12.5 ± 1.3	12.4 ± 1.5	0.37^b^	13.6 ± 1.6	13.1 ± 1.7	< 0.001*^b^
Age at menopause (years)						
≤ 49				43 (28.7%)	160 (26.6%)	0.61^a^
≥ 50				107 (71.3%)	442 (73.4%)	
Mean ± SD				50.8 ± 3.8	50.6 ± 3.5	0.51^b^
Pregnancy (number)						
0	34 (33.0%)	67 (22.1%)	0.003*^a^	25 (16.7%)	75 (12.5%)	0.40^a^
1	24 (23.3%)	46 (15.2%)		15 (10.0%)	62 (10.3%)	
≥ 2	45 (43.7%)	190 (62.7%)		110 (73.3%)	465 (77.2%)	
Mean ± SD	1.6 ± 1.5	1.9 ± 1.5	0.049*^b^	2.2 ± 1.4	2.3 ± 1.4	0.64^b^
Parity						
Yes	60 (58.3%)	222 (73.3%)	0.004*^a^	121 (80.7%)	507 (84.2%)	0.29^a^
No	43 (41.7%)	81 (26.7%)		29 (19.3%)	95 (15.8%)	
Age at primiparity (years)						
Nulliparity	43 (41.7%)	81 (26.7%)	0.02*^a^	29 (19.3%)	95 (15.8%)	0.63^a^
≤ 24	14 (13.6%)	40 (13.2%)		39 (26.0%)	182 (30.2%)	
25–29	27 (26.2%)	91 (30.0%)		60 (40.0%)	242 (40.2%)	
≥ 30	19 (18.4%)	91 (30.0%)		22 (14.7%)	83 (13.8%)	
Mean ± SD	28.1 ± 4.2	28.5 ± 4.5	0.50^b^	26.3 ± 4.0	26.2 ± 3.8	0.75^b^
Breastfeeding						
Yes	54 (52.4%)	215 (71.0%)	0.001*^a^	105 (70.0%)	481 (79.9%)	0.009*^a^
No	49 (47.6%)	88 (29.0%)		45 (30.0%)	121 (20.1%)	
Benign breast disease						
Yes	16 (15.5%)	30 (9.9%)	0.12^a^	18 (12.0%)	103 (17.1%)	0.13^a^
No	87 (84.5%)	273 (90.1%)		132 (88.0%)	499 (82.9%)	
Family history of breast cancer						
Yes	23 (22.3%)	41 (13.5%)	0.034*^a^	21 (14.0%)	94 (15.6%)	0.62^a^
No	80 (77.7%)	262 (86.5%)		129 (86.0%)	508 (84.4%)	
Hyperlipidemia						
Yes				31 (20.7%)	54 (9.0%)	< 0.001*^a^
No				119 (79.3%)	548 (91.0%)	
Diabetes mellitus						
Yes				9 (6.0%)	15 (2.5%)	0.029*^a^
No				141 (94.0%)	587 (97.5%)	

^a^Chi-squared test. ^b^Student’s *t*-test. *P-values < 0.05 are considered significant.

### Serum levels of testosterone and 25-hydroxyvitamin D

Serum levels of testosterone (mean ± SD) were significantly higher in patients than in controls in both pre- and postmenopausal women (*P* = 0.04 and *P* = 0.001, respectively, Table 2). In contrast, serum levels of 25-hydroxyvitamin D (mean ± SD) were significantly lower in patients compared to controls in both pre- and postmenopausal women (*P* = 0.005 and *P* < 0.001, respectively, Table 2). When analyzed in categorized evaluation using the cut-off, higher levels of serum testosterone and lower levels of serum 25-hydroxyvitamin D in patients compared to controls were confirmed in both pre- and postmenopausal women (Table 2).

**Table 2:** Serum levels of testosterone and 25-hydroxyvitamin D between patients and controls

	Patients	Controls	*P*-values
Premenopausal women	*n* = 103	*n* = 303	
Testosterone (ng/ml)			
< 0.16	38 (36.9%)	157 (51.8%)	0.009*^a^
≥ 0.16	65 (63.1%)	146 (48.2%)	
Mean ± SD	0.19 ± 0.11	0.17 ± 0.10	0.04*^b^
25-hydroxyvitamin D (ng/ml)			
< 20.0	71 (68.9%)	174 (57.4%)	0.039*^a^
≥ 20.0	32 (31.1%)	129 (42.6%)	
Mean ± SD	17.04 ± 6.46	19.04 ± 6.16	0.005*^b^
Postmenopausal women	*n* = 150	*n* = 602	
Testosterone (ng/ml)			
< 0.13	52 (34.7%)	297 (49.3%)	0.001*^a^
≥ 0.13	98 (65.3%)	305 (50.7%)	
Mean ± SD	0.19 ± 0.13	0.15 ± 0.10	0.001*^b^
25-hydroxyvitamin D (ng/ml)			
< 20.0	68 (45.3%)	123 (20.4%)	< 0.001*^a^
≥ 20.0	82 (54.7%)	479 (79.6%)	
Mean ± SD	20.96 ± 6.91	25.73 ± 7.15	< 0.001*^b^

^a^Chi-squared test. bStudent’s *t*-test. *P-values < 0.05 are considered significant.

### Common genetic variants

Risk allele frequencies of rs3803662 (*P* = 0.004), rs2046210 (*P* < 0.001), rs4784227 (*P* = 0.049), and rs4973768 (*P* = 0.043) in premenopausal women, and risk allele frequencies of rs2046210 (*P* < 0.001) and rs4784227 (*P* = 0.044) in postmenopausal women were significantly higher in patients compared with those in controls (Table 3). In premenopausal women, four SNPs, TOX3-rs3803662 (odds ratio (OR) = 3.48, 95% confidence interval (CI) 1.63–7.44; *P* = 0.001 in the dominant model), ESR1-rs2046210 (OR = 2.16, 95% CI 1.33–3.49; *P* = 0.002 in the dominant model), 8q24-rs13281615 (OR = 1.74, 95% CI 1.07–2.81; *P* = 0.025 in the recessive model), and SLC4A7-rs4973768 (OR = 5.46, 95% CI 1.62–18.35; *P* = 0.006 in the recessive model), showed significant association with increased risk of ER-positive breast cancer (Table 4). On the other hand, two SNPs, TOX3-rs3803662 (OR = 1.53, 95% CI 1.04–2.26; *P* = 0.031 in the recessive model) and ESR1-rs2046210 (OR = 1.96, 95% CI 1.33–2.87; *P* = 0.001 in the dominant model), showed a significant association with increased risk of ER-positive breast cancer in postmenopausal women (Table 4).

**Table 3:** Allele frequencies of SNPs in pre- and postmenopausal women

Genomic loci		Premenopausal women			Postmenopausal women		
Gene/location		Patients (%)	Controls (%)	*P*-values^a^	Patients (%)	Controls (%)	*P*-values^a^
rs3803662 (C>T)	CC	9 (8.7%)	69 (22.8%)	0.004*	24 (16.0%)	135 (22.4%)	0.62
TOX3/16q12	CT	57 (55.3%)	157 (51.8%)		73 (48.7%)	306 (50.8%)	
	TT	37 (35.9%)	77 (25.4%)		53 (35.3%)	161 (26.7%)	
rs2046210 (C>T)	CC	34 (33.0%)	161 (53.1%)	< 0.001*	50 (33.3%)	293 (48.7%)	< 0.001*
ESR1/6q25	CT	48 (46.6%)	114 (37.6%)		66 (44.0%)	256 (42.5%)	
	TT	21 (20.4%)	28 (9.2%)		34 (22.7%)	53 (8.8%)	
rs13281615 (A>G)	AA	18 (17.5%)	60 (19.8%)	0.16	21 (14.0%)	86 (14.3%)	0.81
Unknown/8q24	AG	42 (40.8%)	148 (48.8%)		72 (48.0%)	304 (50.5%)	
	GG	43 (41.7%)	95 (31.4%)		57 (38.0%)	212 (35.2%)	
							
rs4784227 (C>T)	CC	53 (51.5%)	188 (62.0%)	0.049*	71 (47.3%)	353 (58.6%)	0.044*
LOC643714/6q12	CT	40 (38.8%)	102 (33.7%)		70 (46.7%)	219 (36.4%)	
	TT	10 (9.7%)	13 (4.3%)		9 (6.0%)	30 (5.0%)	
							
rs4973768 (C>T)	CC	61 (59.2%)	201 (66.3%)	0.043*	94 (62.7%)	401 (66.6%)	0.50
SLC4A7/3p24	CT	35 (34.0%)	96 (31.7%)		50 (33.3%)	172 (28.6%)	
	TT	7 (6.8%)	6 (2.0%)		6 (4.0%)	29 (4.8%)	
rs10046 (C>T)	CC	32 (31.1%)	104 (34.3%)	0.35			
CYP19A1/5q21	CT	53 (51.5%)	132 (43.6%)				
	TT	18 (17.5%)	67 (22.1%)				
rs743572 (A>G)	AA	28 (27.2%)	81 (26.7%)	0.60			
CYP17A1/10q24	AG	48 (46.6%)	156 (51.5%)				
	GG	27 (26.2%)	66 (21.8%)				
rs1042522 (C>G)	CC				62 (41.3%)	237 (39.4%)	0.86
TP53/17p13	CG				65 (43.3%)	276 (45.8%)	
	GG				23 (15.3%)	89 (14.8%)	
rs2583506 (C>T)	CC				118 (78.7%)	504 (83.7%)	0.27
TSPYL5/8q22	CT				29 (19.3%)	92 (15.3%)	
	TT				3 (2.0%)	6 (1.0%)	

^a^Chi-squared test. *P-values < 0.05 are considered significant.

**Table 4:** SNPs with significant associations with ER-positive breast cancer risk

	Premenopausal women					Postmenopausal women		
	Patients (%)	Controls (%)	OR (95% CI)	*P*-values^a^	Patients (%)	Controls (%)	OR (95% CI)	*P*-values^a^
TOX3/16q12-rs3803662 (C>T)								
Co-dominant CC	9 (8.7%)	69 (22.8%)	1 (ref.)	0.005*	24 (16.0%)	135 (22.4%)	1 (ref.)	0.05
CT	57 (55.3%)	157 (51.8%)	3.27		73 (48.7%)	306 (50.8%)	1.38	
			(1.50–7.16)				(0.82–2.30)	
TT	37 (35.9%)	77 (25.4%)	3.86		53 (35.3%)	161 (26.7%)	1.94	
			(1.69–8.81)				(1.12–3.35)	
Dominant CC	9 (8.7%)	69 (22.8%)	1 (ref.)	0.001*	24 (16.0%)	135 (22.4%)	1 (ref.)	0.08
CT+TT	94 (91.3%)	234 (77.2%)	3.48		126 (84.0%)	467 (77.6%)	1.57	
			(1.63–7.44)				(0.96–2.54)	
Recessive CC+CT	66 (64.1%)	226 (74.6%)	1 (ref.)	0.09	97 (64.7%)	441 (73.3%)	1 (ref.)	0.031*
TT	37 (35.9%)	77 (25.4%)	1.48		53 (35.3%)	161 (26.7%)	1.53	
			(0.90–2.44)				(1.04–2.26)	
ESR1/6q25-rs2046210 (C>T)								
Co-dominant CC	34 (33.0%)	161 (53.1%)	1 (ref.)	0.002*	50 (33.3%)	293 (48.7%)	1 (ref.)	< 0.001*
CT	48 (46.6%)	114 (37.6%)	1.89		66 (44.0%)	256 (42.5%)	1.55	
			(1.13–3.17)				(1.03–2.34)	
TT	21 (20.4%)	28 (9.2%)	3.22		34 (22.7%)	53 (8.8%)	4.01	
			(1.59–6.53)				(2.33–6.89)	
Dominant CC	34 (33.0%)	161 (53.1%)	1 (ref.)	0.002*	50 (33.3%)	293 (48.7%)	1 (ref.)	0.001*
CT+TT	69 (67.0%)	142 (46.9%)	2.16		100 (66.7%)	309 (51.3%)	1.96	
			(1.33–3.49)				(1.33–2.87)	
Recessive CC+CT	82 (79.6%)	275 (90.8%)	1 (ref.)	0.011*	116 (77.3%)	549 (91.2%)	1 (ref.)	< 0.001*
TT	21 (20.4%)	28 (9.2%)	2.34		34 (22.7%)	53 (8.8%)	3.20	
			(1.22–4.48)				(1.96–5.23)	
8q24-rs13281615 (A>G)								
Co-dominant AA	18 (17.5%)	60 (19.8%)	1 (ref.)	0.07				
AG	42 (40.8%)	148 (48.8%)	0.77					
			(0.41–1.46)					
GG	43 (41.7%)	95 (31.4%)	1.46					
			(0.76–2.80)					
Dominant AA	18 (17.5%)	60 (19.8%)	1 (ref.)	0.80				
AG+GG	85 (82.5%)	243 (80.2%)	1.02					
			(0.57–1.84)					
Recessive AA+AG	60 (58.3%)	208 (68.6%)	1 (ref.)	0.025*				
GG	43 (41.7%)	95 (31.4%)	1.74					
			(1.07–2.81)					
SLC4A7/3p24-rs4973768 (C>T)								
Co-dominant CC	61 (59.2%)	201 (66.3%)	1 (ref.)	0.018*				
CT	35 (34.0%)	96 (31.7%)	1.21					
			(0.74–2.00)					
TT	7 (6.8%)	6 (2.0%)	5.83					
			(1.71–19.88)					
Dominant CC	61 (59.2%)	201 (66.3%)	1 (ref.)	0.16				
CT+TT	42 (40.8%)	102 (33.7%)	1.41					
			(0.88–2.27)					
Recessive CC+CT	96 (93.2%)	297 (98.0%)	1 (ref.)	0.006*				
TT	7 (6.8%)	6 (2.0%)	5.46					
			(1.62–18.35)					

OR, odds ratio; CI, confidence interval; ref., reference ^a^Logistic regression analysis.

*P-values < 0.05 are considered significant.

### Creating risk prediction models

**Figure 1:**
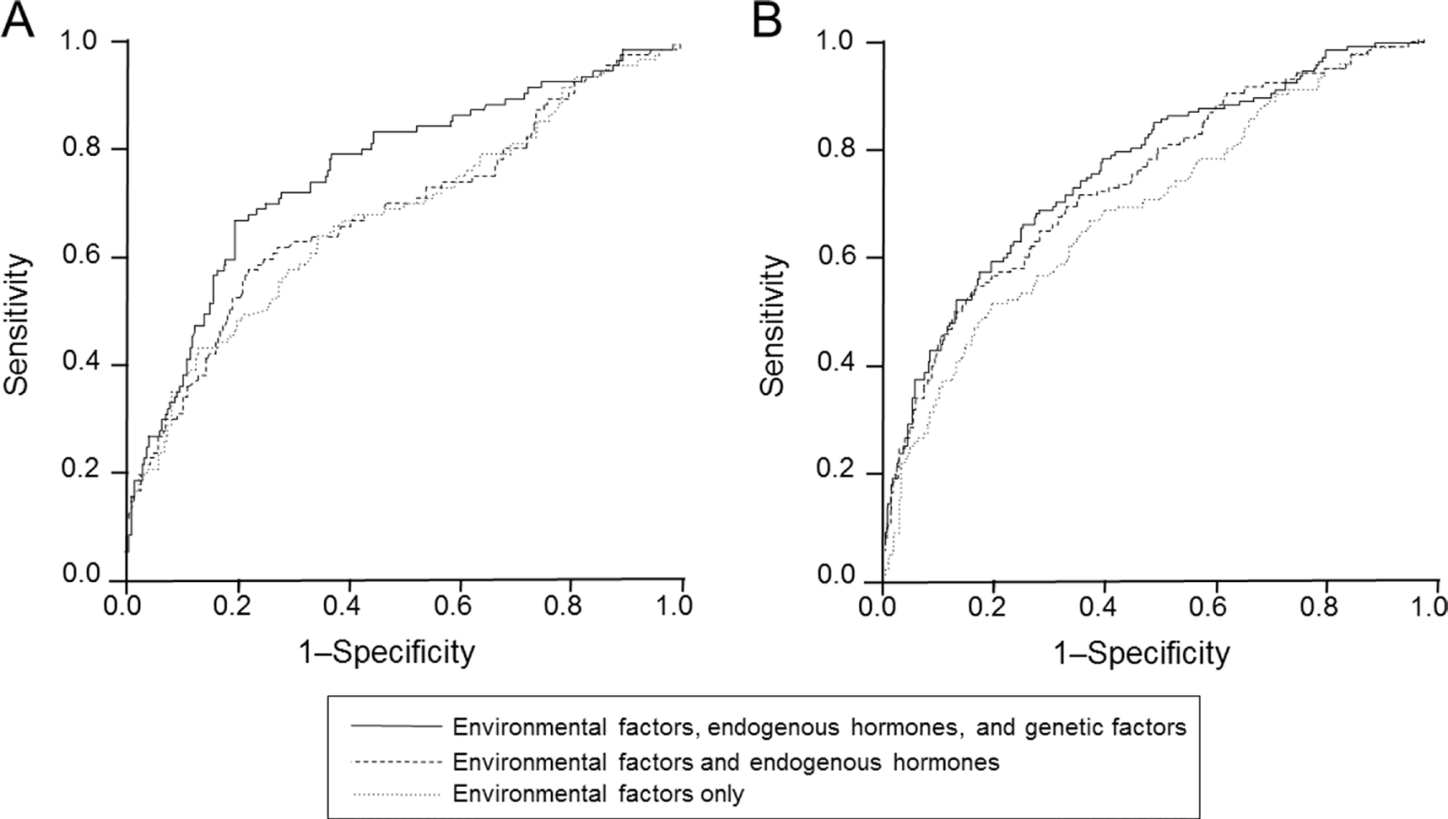
ROC curves for three different models for premenopausal (**A**) and postmenopausal (**B**) women. The upper black lines represent the risk model including environmental factors, endogenous hormones, and genetic factors.

We first created the receiver-operating characteristic (ROC) curves with the area under the curves (AUCs) for three different models. One model took into account environmental factors only, the second model included both environmental factors and endogenous hormones, and the third model included all factors including genetic factors (Figure 1). The risk model of environmental factors only showed the smallest AUCs, with 0.708 for premenopausal women (Figure 1A) and 0.693 for postmenopausal women (Figure 1B). The AUCs of the model including both environmental factors and endogenous hormones were 0.716 for premenopausal women (Figure 1A) and 0.745 for postmenopausal women (Figure 1B). The model including all the factors including genetic factors showed the largest AUCs; 0.785 for premenopausal women (Figure 1A) and 0.764 for postmenopausal women (Figure 1B).

**Figure 2:**
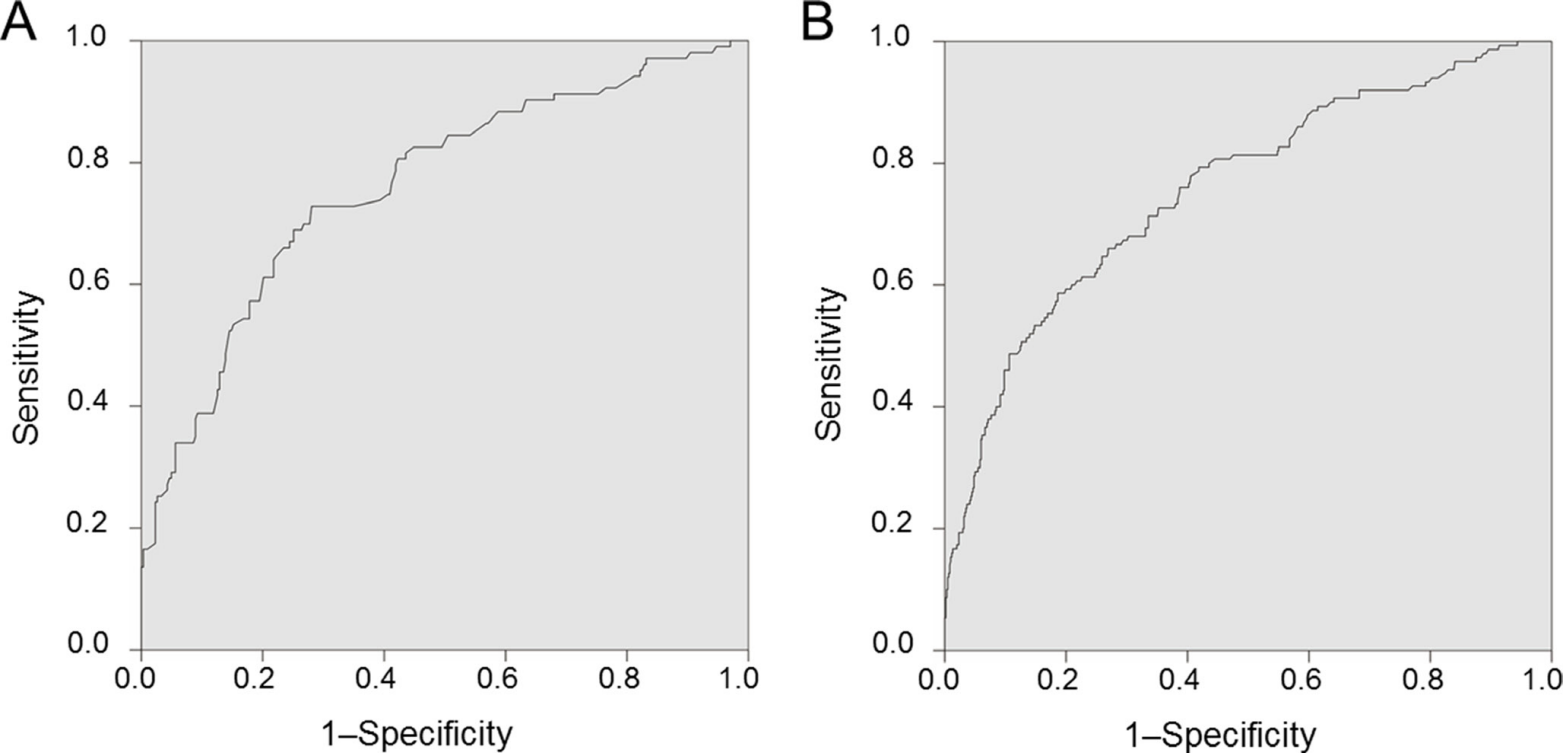
**(A)** ROC curve of the best risk model for premenopausal women. The AUC is estimated as 0.762. **(B)** ROC curve of the best risk model for postmenopausal women. The AUC is estimated as 0.757.

The best risk prediction models with the most effective risk factors were established using the backward stepwise selection method. Finally, the following factors were included in the best risk prediction models: a history of breastfeeding, a history of benign breast disease, CT+TT at TOX3-rs3803662, CT+TT at ESR1-rs2046210, GG at 8q24-rs13281615, and TT at SLC4A7-rs4973768 for premenopausal women with an AUC of 0.762 (Table 5 and Figure 2A), and BMI, a history of breastfeeding, hyperlipidemia, serum testosterone levels, serum 25-hydroxyvitamin D levels, TT at TOX3-rs3803662, and CT+TT at ESR1-rs2046210 for postmenopausal women with an AUC of 0.757 (Table 5 and Figure 2B).

**Table 5:** Factors of the best risk prediction models for pre- and postmenopausal women

	OR	95% CI	***P*-values**^a^
Premenopausal women			
Breastfeeding (yes)	0.48	0.29–0.81	0.006
Benign breast disease (yes)	2.37	1.13–4.99	0.023
TOX3-rs3803662 (CT+TT)	4.25	1.86–9.71	0.001
ESR1-rs2046210 (CT+TT)	2.00	1.20–3.34	0.008
8q24-rs13281615 (GG)	1.82	1.09–3.04	0.022
SLC4A7-rs4973768 (TT)	6.83	1.93–24.16	0.003
Postmenopausal women			
BMI (≥ 25 kg/m^2^)	1.83	1.17–2.86	0.009
Breastfeeding (yes)	0.47	0.30–0.73	0.001
Hyperlipidemia (yes)	2.07	1.19–3.61	0.010
Serum testosterone levels (≥ 0.13 ng/mL)	1.83	1.22–2.75	0.003
Serum 25-hydroxyvitamin D levels (≥ 20 ng/mL)	0.29	0.19–0.44	< 0.001
TOX3-rs3803662 (TT)	1.74	1.14–2.64	0.010
ESR1-rs2046210 (CT+TT)	1.90	1.26–2.87	0.002

OR, odds ratio; CI, confidence interval. ^a^Logistic regression analysis with adjustment for age.

## DISCUSSION

To identify important risk predictors, we created risk prediction models for ER-positive, HER2-negative breast cancer in Japanese women. In this study, all of the patients were newly diagnosed since 2011, and control women were recruited in 2015. Because both patients and controls who participated in this study were recently diagnosed or recruited, this study is able to reflect the recent notable increase of incidence of ER-positive breast cancer in Japanese women. Moreover, the quality of this study has been much improved compared with our previous study [9], because three to four times as many control women compared with patients participated in the present study. Furthermore, we analyzed recently-reported genetic predictors and serum vitamin D, in addition to the predictive factors that we reported in our previous study. Indeed, all four SNPs that were included in our best models were recently-reported genetic variants identified as risk predictors in Japanese women [10]. In our best risk prediction models, two environmental factors and four genetic variants were included for premenopausal women, whereas three environmental factors, two endogenous factors, and two genetic variants were included for postmenopausal women. It is possible that younger women, such as premenopausal women, might be more likely to be affected by genetic factors for development of ER-positive breast cancer, whereas environmental factors might be critical for the development of ER-positive breast cancer in postmenopausal women.

A history of never breastfeeding, and a positive history of benign breast disease were identified as environmental risk factors for premenopausal women, while a history of never breastfeeding, higher BMI, and the presence of hyperlipidemia were environmental risk factors for postmenopausal women in our analysis. These environmental factors are established risk factors for breast cancer [8]. A history of breastfeeding and BMI were included as risk factors in the Gail model, which is one of the breast cancer risk assessment tools. Chlebowski and colleagues demonstrated that the Gail model identified populations at increasing risk for ER-positive but not ER-negative breast cancers in postmenopausal women [15]. Breastfeeding is strongly associated with breast cancer risk in both pre- and postmenopausal women [15, 16]. The Ministry of Health, Labor and Welfare in Japan reported that the total birth rate has decreased since the 1970s from an estimated 2.05 in 1974 to an estimated 1.37 in 2008 (http://www.mhlw.go.jp/english/database/db-hw/FY2010/live_births.html). The decline in the birthrate according to recent changes of lifestyle might be the main cause of the recent notable increase in the incidence of ER-positive breast cancer in Japanese women [17].

In addition to environmental predictors, we demonstrated that higher serum testosterone levels and lower serum 25-hydroxyvitamin D levels were observed in both pre- and postmenopausal breast cancer patients compared to those in controls, and these two factors are included in our best risk prediction model for postmenopausal women. We and others previously reported the association of serum testosterone levels with increased risk of ER-positive breast cancer [9, 18, 19]. On the other hand, 25-hydroxyvitamin D, which serves as the pool of biologically active vitamin D, is the indicator of overall vitamin D status. Experimental and epidemiological studies have suggested a potential anti-cancer effect of vitamin D [20–24].

Two genetic variants, TOX3-rs3803662 and ESR1-rs2046210, are included in our best risk prediction models for both pre- and postmenopausal women, and 8q24-rs13281615 and SLC4A7-rs4973768 are included for premenopausal women only. Previous studies reported that rs3803662 were associated with ER-positive breast cancer risk [25], and that all four SNPs were associated with breast cancer risk in Japanese and/or Asian women [10, 25–27]. Because genetic factors correlated with breast cancer risk differ according to ER status and ethnicity, the genetic variants in our models might be risk predictors for ER-positive breast cancer in Japanese women. Furthermore, functional analyses of these genetic variants could lead to identification of the mechanisms of development of ER-positive breast cancer.

There are several limitations to this study. First, this is a case–control study, and therefore some self-reported lifestyle factors may have been uncertain. However, our study might reflect the recent marked increase in the incidence of ER-positive breast cancer, because all of the patients and controls who participated in this study were very recently diagnosed. Second, this study was non-age matched. Third, smoking and alcohol intake, which are considered as environmental risk factors, could not be analyzed, because of difficulties of evaluation and lack of reliability. Fourth, we recruited control women who visited Hokkaido Cancer Society for breast cancer screening. Because Hokkaido is located in the northern part of Japan, backgrounds and environmental factors might not be completely similar to those of the common population of Japanese women.

In conclusion, we created risk prediction models for ER-positive, HER2-negative breast cancer for pre- and postmenopausal Japanese women to identify important risk predictors. Our results suggest that the decline in the birthrate might be the main cause of the recent notable increase in the incidence of ER-positive breast cancer in Japanese women. Inclusion of common genetic variants and serum hormone measurements as well as environmental factors might improve risk assessment models.

## MATERIALS AND METHODS

### Subjects

The study population comprised 253 consecutive Japanese women (103 premenopausal and 150 postmenopausal) aged 40 years or older with ER-positive, HER2-negative breast cancer, both invasive and non-invasive cancers, which were newly diagnosed at Hokkaido University Hospital and Kumamoto University Hospital between January 2011 and December 2014, and 905 control Japanese women (303 premenopausal and 602 postmenopausal) who visited Hokkaido Cancer Society for breast cancer screening consecutively between January and October 2015 and confirmed to be without disease, giving a 1:3 case: control ratio in premenopausal women and a 1:4 case: control ratio in postmenopausal women. Women aged 80 years or older were excluded, because very old women rarely undergo a breast cancer screening. The protocol of this study was approved by the Institutional review committees and conformed to the guidelines of the 1996 Declaration of Helsinki. Family history of breast cancer was defined as positive if first and/or second-degree relatives had had breast cancer. Blood samples from patients were taken before treatment. ER status of the breast cancer tissues was assessed by immunohistochemistry, and tumors with ≥ 1% positive cells were considered positive. Patients with HER2-positive tumors were excluded from this study. Postmenopause was defined as the existence of amenorrhea for more than one year together with low serum levels of estradiol. Women with high levels of serum estradiol were considered premenopausal regardless of whether they had experienced amenorrhea for more than one year.

### Measurement of serum samples

Blood samples were centrifuged at 1300 *g* for 10 min at 15°C, and the separated sera were stored at −80°C. Concentrations of testosterone and 25-hydroxyvitamin D, which are recently-reported predictive factors and able to evaluate commonly, were measured by commercially available immunoassays. Serum testosterone levels were measured by electro-chemiluminescence immunoassay using Ecrusis Testosterone (Roche Diagnostics, Tokyo, Japan). Serum levels of 25-hydroxyvitamin D were measured by direct radioimmunoassay using 25-Hydroxyvitamin D 125I RIA Kit (DiaSorin Inc, Stillwater, MN, USA). The concentrations of the two factors were stratified using a cut-off for categorized evaluation; the median was used for testosterone, and a threshold of vitamin D deficiency (20 ng/mL) was used for 25-hydroxyvitamin D levels [28].

### Genotyping

Genomic DNA was extracted from the blood samples with a QIAamp DNA blood mini kit (QIAQEN, Tokyo Japan). Nine genetic variants reported as risk predictors for ER-positive breast cancer or found in Asian women were analyzed: TOX3-rs3803662 [10, 25, 26, 29], ESR1-rs2046210 [10, 25, 27], 8q24-rs13281615 [10], LOC643714-rs4784227 [10, 30], and SLC4A7-rs4973768 [10, 25] for all women, CYP19A1-rs10046 [9] and CYP17A1-rs743572 [9] for premenopausal women only, and TP53-rs1042522 [9] and TSPYL5-rs2583506 [31] for postmenopausal women only. Genotyping was carried out using TaqMan Single Nucleotide Polymorphism Genotyping Assays on the StepOnePlus Real-Time PCR System (Applied Biosystems, Foster City, CA, USA).

### Statistical analyses

Differences in continuous variables between patients and controls were evaluated by Student’s *t*-test, and categorical variables were analyzed by the Chi-squared test. Odds ratios with 95% confidence intervals were calculated to assess the strength of influence of each SNP on breast cancer risk using logistic regression models after adjustment for age. A co-dominant model, a dominant model, and a recessive model of risk alleles were established, and the SNPs showing a significant association with risk were selected in multivariate analyses. Allele frequencies of all the nine SNPs in controls were verified to be in line with Hardy–Weinberg equilibrium by the Chi-squared test. Multivariate binary logistic regression analyses were performed for environmental factors only, environmental factors and endogenous factors, and all factors including SNPs. ROC curves were generated with the AUC for the three models to evaluate the models with different variables. Finally, the best model with an ROC curve calculated by the essential factors was generated using the backward stepwise selection method. All statistical analyses were carried out using IBM SPSS Statistics 22.0 (IBM Corp., Armonk, NY, USA). *P* values of < 0.05 were considered statistically significant.

## Abbreviations

ER: estrogen receptor
HER2: human epidermal growth factor receptor type 2
SNP: single nucleotide polymorphism
ROC: receiver-operating characteristic
AUC: area under the curve

## References

[1] Yamashita H, Iwase H, Toyama T, Takahashi S, Sugiura H, Yoshimoto N, Endo Y, Fujii Y, Kobayashi S (2011). Estrogen receptor-positive breast cancer in Japanese women: trends in incidence, characteristics, and prognosis. Ann Oncol.

[2] Kurebayashi J, Miyoshi Y, Ishikawa T, Saji S, Sugie T, Suzuki T, Takahashi S, Nozaki M, Yamashita H, Tokuda Y, Nakamura S (2015). Clinicopathological characteristics of breast cancer and trends in the management of breast cancer patients in Japan: Based on the Breast Cancer Registry of the Japanese Breast Cancer Society between 2004 and 2011. Breast Cancer.

[3] Goss PE, Ingle JN, Ales-Martinez JE, Cheung AM, Chlebowski RT, Wactawski-Wende J, McTiernan A, Robbins J, Johnson KC, Martin LW, Winquist E, Sarto GE, Garber JE (2011). Exemestane for breast-cancer prevention in postmenopausal women. N Engl J Med.

[4] Cuzick J, Sestak I, Forbes JF, Dowsett M, Knox J, Cawthorn S, Saunders C, Roche N, Mansel RE, von Minckwitz G, Bonanni B, Palva T, Howell A (2014). Anastrozole for prevention of breast cancer in high-risk postmenopausal women (IBIS-II): an international, double-blind, randomised placebo-controlled trial. Lancet.

[5] Nelson HD, Smith ME, Griffin JC, Fu R (2013). Use of medications to reduce risk for primary breast cancer: a systematic review for the U.S. Preventive Services Task Force. Ann Intern Med.

[6] Yang XR, Chang-Claude J, Goode EL, Couch FJ, Nevanlinna H, Milne RL, Gaudet M, Schmidt MK, Broeks A, Cox A, Fasching PA, Hein R, Spurdle AB (2011). Associations of breast cancer risk factors with tumor subtypes: a pooled analysis from the Breast Cancer Association Consortium studies. J Natl Cancer Inst.

[7] Garcia-Closas M, Chanock S (2008). Genetic susceptibility loci for breast cancer by estrogen receptor status. Clin Cancer Res.

[8] Taira N, Arai M, Ikeda M, Iwasaki M, Okamura H, Takamatsu K, Yamamoto S, Ohsumi S, Mukai H (2015). Japanese Breast Cancer S. The Japanese Breast Cancer Society clinical practice guideline for epidemiology and prevention of breast cancer. Breast Cancer.

[9] Yoshimoto N, Nishiyama T, Toyama T, Takahashi S, Shiraki N, Sugiura H, Endo Y, Iwasa M, Fujii Y, Yamashita H (2011). Genetic and environmental predictors, endogenous hormones and growth factors, and risk of estrogen receptor-positive breast cancer in Japanese women. Cancer Sci.

[10] Sueta A, Ito H, Kawase T, Hirose K, Hosono S, Yatabe Y, Tajima K, Tanaka H, Iwata H, Iwase H, Matsuo K (2012). A genetic risk predictor for breast cancer using a combination of low-penetrance polymorphisms in a Japanese population. Breast Cancer Res Treat.

[11] Zheng W, Wen W, Gao YT, Shyr Y, Zheng Y, Long J, Li G, Li C, Gu K, Cai Q, Shu XO, Lu W (2010). Genetic and clinical predictors for breast cancer risk assessment and stratification among Chinese women. J Natl Cancer Inst.

[12] Wacholder S, Hartge P, Prentice R, Garcia-Closas M, Feigelson HS, Diver WR, Thun MJ, Cox DG, Hankinson SE, Kraft P, Rosner B, Berg CD, Brinton LA (2010). Performance of common genetic variants in breast-cancer risk models. N Engl J Med.

[13] Anderson WF, Jatoi I, Sherman ME (2009). Qualitative age interactions in breast cancer studies: mind the gap. J Clin Oncol.

[14] Yamashita H (2015). Tumor biology in estrogen receptor-positive, human epidermal growth factor receptor type 2-negative breast cancer: Mind the menopausal status. World J Clin Oncol.

[15] Chlebowski RT, Anderson GL, Lane DS, Aragaki AK, Rohan T, Yasmeen S, Sarto G, Rosenberg CA, Hubbell FA (2007). Predicting risk of breast cancer in postmenopausal women by hormone receptor status. J Natl Cancer Inst.

[16] Collaborative Group on Hormonal Factors in Breast Cancer (2002). Breast cancer and breastfeeding: collaborative reanalysis of individual data from 47 epidemiological studies in 30 countries, including 50 302 women with breast cancer and 96 973 women without the disease. Lancet.

[17] Kobayashi S, Sugiura H, Ando Y, Shiraki N, Yanagi T, Yamashita H, Toyama T (2012). Reproductive history and breast cancer risk. Breast Cancer.

[18] Tworoger SS, Zhang X, Eliassen AH, Qian J, Colditz GA, Willett WC, Rosner BA, Kraft P, Hankinson SE (2014). Inclusion of endogenous hormone levels in risk prediction models of postmenopausal breast cancer. J Clin Oncol.

[19] Fortner RT, Eliassen AH, Spiegelman D, Willett WC, Barbieri RL, Hankinson SE (2013). Premenopausal endogenous steroid hormones and breast cancer risk: results from the Nurses' Health Study II. Breast Cancer Res.

[20] Feldman D, Krishnan AV, Swami S, Giovannucci E, Feldman BJ (2014). The role of vitamin D in reducing cancer risk and progression. Nat Rev Cancer.

[21] Wang D, Velez de-la-Paz OI, Zhai JX, Liu DW (2013). Serum 25-hydroxyvitamin D and breast cancer risk: a meta-analysis of prospective studies. Tumour Biol.

[22] Kim Y, Je Y (2014). Vitamin D intake, blood 25(OH)D levels, and breast cancer risk or mortality: a meta-analysis. Br J Cancer.

[23] Reimers LL, Crew KD, Bradshaw PT, Santella RM, Steck SE, Sirosh I, Terry MB, Hershman DL, Shane E, Cremers S, Dworakowski E, Teitelbaum SL, Neugut AI (2015). Vitamin D-related gene polymorphisms, plasma 25-hydroxyvitamin D, and breast cancer risk. Cancer Causes Control.

[24] Shirazi L, Almquist M, Borgquist S, Malm J, Manjer J (2016). Serum vitamin D (25OHD3) levels and the risk of different subtypes of breast cancer: A nested case-control study. Breast.

[25] Han W, Woo JH, Yu JH, Lee MJ, Moon HG, Kang D, Noh DY (2011). Common genetic variants associated with breast cancer in Korean women and differential susceptibility according to intrinsic subtype. Cancer Epidemiol Biomarkers Prev.

[26] Low SK, Takahashi A, Ashikawa K, Inazawa J, Miki Y, Kubo M, Nakamura Y, Katagiri T (2013). Genome-wide association study of breast cancer in the Japanese population. PLoS One.

[27] Cai Q, Wen W, Qu S, Li G, Egan KM, Chen K, Deming SL, Shen H, Shen CY, Gammon MD, Blot WJ, Matsuo K, Haiman CA (2011). Replication and functional genomic analyses of the breast cancer susceptibility locus at 6q25.1 generalize its importance in women of chinese, Japanese, and European ancestry. Cancer Res.

[28] Holick MF, Binkley NC, Bischoff-Ferrari HA, Gordon CM, Hanley DA, Heaney RP, Murad MH, Weaver CM (2011). Evaluation, treatment, and prevention of vitamin D deficiency: an Endocrine Society clinical practice guideline. J Clin Endocrinol Metab.

[29] Easton DF, Pooley KA, Dunning AM, Pharoah PD, Thompson D, Ballinger DG, Struewing JP, Morrison J, Field H, Luben R, Wareham N, Ahmed S, Healey CS (2007). Genome-wide association study identifies novel breast cancer susceptibility loci. Nature.

[30] Long J, Cai Q, Shu XO, Qu S, Li C, Zheng Y, Gu K, Wang W, Xiang YB, Cheng J, Chen K, Zhang L, Zheng H (2010). Identification of a functional genetic variant at 16q12.1 for breast cancer risk: results from the Asia Breast Cancer Consortium. PLoS Genet.

[31] Liu M, Ingle JN, Fridley BL, Buzdar AU, Robson ME, Kubo M, Wang L, Batzler A, Jenkins GD, Pietrzak TL, Carlson EE, Goetz MP, Northfelt DW (2013). TSPYL5 SNPs: association with plasma estradiol concentrations and aromatase expression. Mol Endocrinol.

